# Building and Sustaining Public Trust in Health Data Sharing for Musculoskeletal Research: Semistructured Interview and Focus Group Study

**DOI:** 10.2196/53024

**Published:** 2024-10-15

**Authors:** Zainab K Yusuf, William G Dixon, Charlotte Sharp, Louise Cook, Søren Holm, Caroline Sanders

**Affiliations:** 1 Centre for Epidemiology Versus Arthritis University of Manchester Manchester United Kingdom; 2 NIHR School for Primary Care Research University of Manchester Manchester United Kingdom; 3 Manchester Biomedical Research Centre National Institute for Health and Care Research Manchester United Kingdom; 4 The Kellgren Centre for Rheumatology Manchester Royal Infirmary Manchester United Kingdom; 5 Centre for Social Ethics and Policy University of Manchester Manchester United Kingdom; 6 Centre for Primary Care and Health Services Research NIHR Greater Manchester Patient Safety Research Collaboration University of Manchester Manchester United Kingdom

**Keywords:** data sharing, public trust, musculoskeletal, marginalized communities, underserved communities

## Abstract

**Background:**

Although many people are supportive of their deidentified health care data being used for research, concerns about privacy, safety, and security of health care data remain. There is low awareness about how data are used for research and related governance. Transparency about how health data are used for research is crucial for building public trust. One proposed solution is to ensure that affected communities are notified, particularly marginalized communities where there has previously been a lack of engagement and mistrust.

**Objective:**

This study aims to explore patient and public perspectives on the use of deidentified data from electronic health records for musculoskeletal research and to explore ways to build and sustain public trust in health data sharing for a research program (known as “the Data Jigsaw”) piloting new ways of using and analyzing electronic health data. Views and perspectives about how best to engage with local communities informed the development of a public notification campaign about the research.

**Methods:**

Qualitative methods data were generated from 20 semistructured interviews and 8 focus groups, comprising 48 participants in total with musculoskeletal conditions or symptoms, including 3 carers. A presentation about the use of health data for research and examples from the specific research projects within the program were used to trigger discussion. We worked in partnership with a patient and public involvement group throughout the research and cofacilitated wider community engagement.

**Results:**

Respondents were supportive of their health care data being shared for research purposes, but there was low awareness about how electronic health records are used for research. Security and governance concerns about data sharing were noted, including collaborations with external companies and accessing social care records. Project examples from the Data Jigsaw program were viewed positively after respondents knew more about how their data were being used to improve patient care. A range of different methods to build and sustain trust were deemed necessary by participants. Information was requested about: data management; individuals with access to the data (including any collaboration with external companies); the National Health Service’s national data opt-out; and research outcomes. It was considered important to enable in-person dialogue with affected communities in addition to other forms of information.

**Conclusions:**

The findings have emphasized the need for transparency and awareness about health data sharing for research, and the value of tailoring this to reflect current and local research where residents might feel more invested in the focus of research and the use of local records. Thus, the provision for targeted information within affected communities with accessible messages and community-based dialogue could help to build and sustain public trust. These findings can also be extrapolated to other conditions beyond musculoskeletal conditions, making the findings relevant to a much wider community.

## Introduction

### Background

Electronic health records contain information documented during consultations between patients and health care professionals, such as diagnoses, test results, and treatments provided. While primarily used for direct care, these records can be deidentified to support research and planning without explicit individual consent [1], supporting the development of new treatments and services [2-6]. In the United Kingdom, anonymized data from National Health Service (NHS) patients are made available for research with relevant governance approvals. Individuals may opt out of data sharing for research by completing the national opt-out process [7]. Before the COVID-19 pandemic, research suggested that most patients were positive and altruistic regarding the confidential reuse of health data [8], and this has continued to be reflected in post–COVID-19 publications [9]. Greater public confidence has been reported in health care organizations (such as the NHS in the United Kingdom), educational institutions (such as universities), or charitable sectors for reusing health data for research rather than in commercial companies [8-11].

There have been ongoing concerns about the low levels of awareness and understanding about data sharing for research, especially in people from underserved and marginalized communities who are less likely to have been included in discussion and research on this topic. Following previous high-profile debates and scandals regarding health data sharing [12-14], there is a need for greater understanding among the research community of how best to build and sustain public trust, including with diverse communities. Issues such as low awareness, consent preferences, and data security remain key areas of consideration when trying to establish public trust [15-21]. Recent reports have highlighted key variations in levels of trust according to factors such as age, with older people less likely to trust data sharing [22]. In addition, there has been lower trust in data sharing among British ethnic minorities [23,24], leading to a recognition that there is more to be done to enable seldom-heard voices to be a part of public dialogue and governance associated with data sharing [25]. This will be crucial for safeguarding and building trust across diverse communities, especially where people may associate data sharing with personal risks and negative consequences. Such negative consequences may include concern about increased risk of discrimination if sensitive health information is shared, or insurance premiums rising if private companies are provided access to an individual’s data [26-28]. Varied approaches might be needed to tackle these different types of concerns. The link between low levels of awareness and low trust emphasizes the need for designing better ways of communicating and building public trust in affected communities.

### Data Security and Public Trust

With the introduction of the integrated care systems, which join up NHS organizations, local authorities, and other third sector bodies to deliver integrated health and care services [29], there has been a move toward ensuring data are managed securely [30]. The recent review by Goldacre et al [31] recommended moving toward a model of data sharing based on the Secure Data Environment (SDE). The SDE enables researchers and analysts to work within a secure computing environment. SDEs are being implemented across several of the United Kingdom’s health data sharing initiatives, including Health Data Research UK [32], Our Future Health [33], and Genomics England [34]. The Goldacre review stipulates that SDEs may help build public trust through “provable, credible steps” to protect patient privacy, and “by being transparent with everyone about everything that is done” with their data [31]. Protecting patient data is, arguably, a moral imperative and of prime importance when trying to build public trust. There is a need for members of the public to learn about how their personal health records are being used for research and the associated benefits and managed risks, so that they can better understand how their data are kept secure [35].

### Purpose and Research Questions

Transparency and public trust are acknowledged as fundamental to enabling and sustaining health data sharing for service improvement and research [31]. Without consent from the public, potential gains from health research would be put at risk. Previous studies have explored ways to improve transparency and establish public trust in the use of health records for research, focusing on generic and conceptual discussions on data sharing [15,18-21]. Beyond a few specific examples, such as in cancer research [36], there has been less research focused on enabling dialogue about health data sharing for specific disease areas. There is also a lack of research focused on local communities which might include perspectives of those whose data are being used for specific research relevant to their health or health conditions. Therefore, it is important to engage in dialogue with affected communities (local and specific to the disease area) to build and sustain public trust, particularly in underserved and marginalized communities. Understanding the perspectives of people from diverse contexts can help tailor appropriate communication materials and methods.

### This Study

This study fills previous gaps in focusing on the specific disease area of musculoskeletal research using health records, and in adopting an inclusive approach to include underserved and marginalized communities to understand and build public trust for this research. The overall aim of this qualitative study was to explore patient perspectives on health data sharing for musculoskeletal research and to improve communications for building public trust. There were 2 main research questions: (1) What are the attitudes and views of patients and carers toward health data sharing for musculoskeletal research and (2) How can we improve transparency and enable dialogue with local communities about health data sharing for musculoskeletal research to build and sustain public trust?

## Methods

### Patient and Public Involvement and Engagement Group

The research team formed a patient and public involvement and engagement (PPIE) group comprising patients with musculoskeletal conditions and leads from relevant community and voluntary sector organizations (n=5). The aim of working with this group was to enable public involvement in all stages of the qualitative research and the coproduction of communications materials to inform patients and public of research being conducted using local health care records. The latter was part of a wider research program known as “the Data Jigsaw” [37]. Authors ZKY and C Sanders met with the PPIE group quarterly to discuss the different aspects of the study, such as the design of participant information sheets, interview and focus group topic guides, and communication materials. They also explained the use of health data to the PPIE group and worked together with them to discuss and refine emerging findings from the interviews and focus groups. A detailed paper focusing on the distinct PPIE aspects will be reported separately.

### Recruitment and Methods

We developed a semistructured interview and focus group topic guide based on themes within previous literature (such as awareness of data sharing, trust, views about potential benefits, and any concerns) and discussions with our PPIE group to explore participants’ views on electronic health data sharing. The topic guide was tested out and refined through multiple meetings with our PPIE group before data collection. The guide started with a brief introduction, followed by open questions about what health data sharing meant to participants, and any prior understanding of health data sharing that they had before joining the study. Initial discussions were based upon their original views and awareness before we presented further information, including a short film to prompt discussion regarding understanding and views about data governance, and project-specific examples delivered as short films by the researchers involved. The latter prompted discussions regarding potential benefits and concerns associated with health data sharing for the musculoskeletal research being undertaken within the local data integration pilot, known as the Data Jigsaw [37]. Discussions were connected to specific research questions being addressed through the database research in the wider Data Jigsaw program (eg, developing an algorithm to detect axial spondyloarthritis, safety and effectiveness of analgesics, and linking health and social care records) [37]. Findings from the initial interviews and focus groups were used to inform co-design of a communications campaign regarding the Data Jigsaw program. Four follow-up focus groups were then conducted to capture perspectives on the communication materials along with discussing general views regarding health data sharing for research (copy of the topic guide used before and after the development of the communications campaign in Multimedia Appendix 1).

Offering participants the choice between taking part in interviews and focus groups allowed people to choose how they would prefer to participate, and provided the study with a combination of different types of data. The interviews provided in-depth patient perspectives. Focus groups enabled open discussion between several participants [38]; generating dialogue about the lack of awareness of health data sharing, security concerns, and privacy of data; as well as ways to improve transparency and awareness of health data sharing in local communities.

We recruited participants, who were not previously known to the researchers, from Greater Manchester, United Kingdom. Participants were recruited using social media platforms and local advertisements in clinical and community settings. All participants met the eligibility criteria if they were >18 years old, able to provide informed consent, had either been medically diagnosed with a musculoskeletal condition or had experience of symptoms (such as chronic pain and joint symptoms), or were carers for people fulfilling those definitions. We used a purposive sample based on whether participants self-identified with the eligibility criteria, and to maximize diversity according to age, gender, ethnicity, and socioeconomic background. We aimed to continue recruitment until identified themes were recurring through iterative data analysis, indicating that data saturation was achieved.

Overall, 8 focus groups were conducted (n=4, 50% before and n=4, 50% after the development of the communications campaign), consisting of 3 to 8 participants each. Focus groups were led by 2 members of the team (C Sanders and ZKY) with extensive expertise in qualitative research. C Sanders is a professor of medical sociology and was the lead facilitator for focus groups. ZKY is an experienced postdoctoral researcher who cofacilitated and took notes throughout the focus groups. Discussions lasted 60 to 90 minutes. We conducted a total of 20 semistructured interviews before the development of the communications campaign, each lasting 30 to 90 minutes. The focus groups and interviews were audio-recorded with consent. Six of the 8 (75%) focus groups were web-based and conducted via Zoom (Zoom Video Communications). The remaining focus groups took place in a local community space in Salford, Greater Manchester. Overall, 19 (95%) interviews were conducted via Zoom (by ZKY) and 1 (5%) interview took place at a local community center (conducted by C Sanders; for completed COREQ [Consolidated Criteria for Reporting Qualitative Research] form [39] for reporting qualitative interviews and focus groups for Multimedia Appendix 2).

### Community Engagement

To enable wider engagement with affected communities, particularly with members of underserved groups, informal discussions took place at local community spaces in Salford, Greater Manchester. This was part of our PPIE strategy to include a diverse range of public and patients. The informal discussions supported wider public dialogue regarding the Data Jigsaw research program [37] and discussed the qualitative research and development of communications for a public notification campaign. These discussions included local members of the public who may not have had the opportunity to express their opinions about health data sharing previously and included people experiencing poverty and homelessness (via a community-based drop-in center), as well as members of the D/deaf community. With the help of the PPIE advisory group, authors ZKY and C Sanders arranged to attend a drop-in center for people at risk of homelessness on 2 occasions to generate interest. Approximately 71 people took part in the informal discussions. Two informal discussions were arranged and took place at the community-based drop-in center with approximately 32 people. Further, ZKY liaised with community gatekeepers to arrange informal discussions with the D/deaf community (15/71) and with volunteers who were part of a community allotment group (24/71).

Informal discussions took place at the same time as the formal focus groups and interviews were being conducted with research participants. The informal discussions were not recorded and no personal data were collected. The ethics committee approved plans for public involvement and wider community engagement. However, formal recorded consent was not required as part of the ethical governance of the study because attendees at informal discussions were not research participants. An introduction to the research topic and the purpose of the discussion was provided at the start of the discussion. It was made clear that the discussion was informal and that we would not be recording any identifiable information and would not be audio-recording the discussions. Similar to the research focus groups, open questions were asked about views and understanding of using health records for research. Brief notes were created during and following the discussions but without any personal identifiers. The notes were in bullet point form to identify prevalent issues. While the informal discussions were not part of the qualitative analysis, we have referred to these where there was resonance with analytic themes. This serves to reflect how public involvement and engagement were embedded throughout (for the completed Guidance for Reporting Involvement of Patients and the Public [GRIPP2] checklist short form [40] refer to Multimedia Appendix 3).

### Ethical Considerations

The study received ethics approval from the NHS London Bloomsbury Research Ethics Committee (21/NW/0354). Participants were informed of the research aims and interests of the study topic using a detailed Patient Information Sheet at least 24 hours in advance of taking part, to enable them to consider participation and address any questions to the research team. The information specified issues that would be discussed during interviews and focus groups, as well as arrangements for audio-recording and use of anonymized data from the transcripts. Participants were informed that they could withdraw from the study at any time (although none did so) and that they could raise any questions or complaints should they wish to. Participants who agreed to be interviewed were asked to provide informed consent before participation. People participating on the web provided verbal consent recorded in advance of the interview or focus group. People participating in a face-to-face interview or focus group provided written consent immediately before the event. At the start of interviews or participation in the focus group, participants were reminded of the purpose of the discussion, and an outline of what would be covered was presented. Assurance of confidentiality and arrangements for the use of anonymized data was provided. Ground rules were outlined at the start of the focus group to support confidentiality and inclusive discussions. We also assured participants that we could pause or stop discussions when requested and give support, should anyone need this. Participants were provided with a £20 (US $26.5) gift shop voucher, bank transfer, or cash, to thank them for their time.

### Data Analysis

Interviews and focus groups were transcribed verbatim by an external supplier and the transcripts were checked by ZKY. Notes were made during the interviews and focus groups and were also used to inform analysis. The data were analyzed thematically, drawing on some techniques of a grounded theory approach, including open coding and constant comparison so that transcripts were continuously compared for the iterative development of key themes [41]. Both C Sanders and ZKY independently read transcripts. Primary open coding on all data was completed by ZKY using NVivo (version 12; Lumivero) to support the coding and organization of the data. C Sanders completed independent coding of 2 initial focus groups to compare and discuss terminology for coding. C Sanders provided reflections and comments on descriptive accounts of coded data from interviews and focus groups throughout. C Sanders and ZKY also met regularly throughout the research to discuss the development and refinement of codes and themes from the data. As noted above, discussions within the interviews and focus groups began with open questions to elicit initial awareness and understanding of health data sharing, before progressing to further discussions focusing on examples of research using health records within the Data Jigsaw program and views about communication needs. This approach inevitably had some bearing on the development of coding, final themes, and elements of both inductive and deductive analysis. Transcripts were not returned to participants for comments. Codes and themes were shared and discussed periodically at wider team meetings, including with the PPIE advisory group. Participants were provided with a summary of the findings at the end of the analysis period and invited to contact the research team with any comments or questions.

## Results

### Participants

Between June 2022 and September 2023, 48 participants took part in the study. Participants were recruited via Twitter (n=10, 21%), Facebook (n=3, 6%), PPIE health research networks (n=11, 23%), local advertisements (n=5, 10%), local community groups (n=9, 19%), and word of mouth (n=1, 2%). Remaining participants (n=9, 19%) contacted the researcher by email after seeing an advert via one of the previous routes (source of advert not specified).

The participant sample included men (21/48, 44%) and women (27/48, 56%) of various ages, ethnic, and socioeconomic backgrounds (Table 1).

**Table 1:** Demographic characteristics of research participants (N=48).

Characteristics		Participants, n (%)
**Diagnosis of a musculoskeletal condition**		
	Yes	44 (92)
	No	4 (8)
**Musculoskeletal symptoms**		
	Chronic pain	37 (77)
	Inflamed joints	36 (75)
	Fatigue	33 (69)
	Loss of mobility and dexterity	36 (75)
	Stiffness	42 (88)
	Other musculoskeletal symptoms	12 (25)
**Age (y)**		
	18-25	4 (8)
	26-35	5 (10)
	36-45	10 (21)
	46-55	5 (10)
	56-65	4 (8)
	66-75	12 (25)
	>75	8 (17)
**Gender**		
	Men	21 (44)
	Women	27 (56)
**Ethnicity**		
	Asian or Asian British	1 (2)
	Black African, Black British, or Caribbean	7 (15)
	Mixed or multiple ethnic groups	8 (17)
	White	32 (67)
**Employment**		
	Employed full-time	14 (29)
	Employed part-time	8 (17)
	Self-employed	2 (4)
	Not employed, looking for work	1 (2)
	Not employed, not looking for work	0 (0)
	Volunteer (unpaid work)	5 (10)
	Student	0 (0)
	Retired	18 (38)

### Findings

The findings are presented in three overarching themes: (1) awareness and motivation for supporting data sharing for musculoskeletal research, (2) issues regarding security and sharing of data for musculoskeletal research beyond the NHS, and (3) transparency and communication needs for building trust in data sharing for musculoskeletal research. The development of 3 broad themes reflected a number of subthemes (Textbox 1), elaborated upon in the following sections.

**Summary of main themes and subthemes.**

**Theme 1: awareness of and motivation for supporting data sharing for musculoskeletal research**

- Understanding of data sharing for research
- Understanding of security and consent for data sharing
- Expectation that data are shared to improve patient care
- Perceived public awareness of data sharing for research
- Limited knowledge of the ability to opt-out
- Altruism and data sharing for the greater good
- Improves patient and safety and benefit
- Patient empowerment

**Theme 2: issues of security and sharing data for musculoskeletal research beyond the National Health Service (NHS)**

- Sharing between NHS and social care
- Stigma and sensitivity of data
- Data breaches
- The role of private companies

**Theme 3: transparency and communication needs for building trust in data sharing for musculoskeletal research**

- Need for simple information
- Need for positive messages and examples
- Need for transparency about security
- Enabling communication of findings from research using health records

### Awareness of and Motivation for Supporting Data Sharing for Musculoskeletal Research

When asked about their views on data sharing, individuals reported a perceived lack of awareness about data sharing for research, not necessarily knowing what it entailed, for example:

I don’t think people know about it as much...I didn’t know...that much about it before I got more involved.

Interview 14, participant # F11

and

[T]hey don’t know it’s happening...

Interview 2, participant # M9

Focus group participants reported a lack of public notifications that raise awareness about data sharing initiatives (with the exception of Cancer Research UK [36]):

[T]here’s nothing...I’ve never seen anything on the walls, or anything like that...the only research I know is cancer research. That’s the only one I know of...there’s nothing on anything else, as far as I know.

Focus group 3, participant # F41

Others agreed, noting a marked lack of awareness. Cancer research may be particularly salient to the public because of the national advertising campaigns by Cancer Research UK [36]. Beyond specific examples like cancer, public notification about health data sharing tends to be generic, possibly reducing public interest. The comparison to other conditions, such as cancer, exemplifies the lack of awareness of data sharing initiatives for musculoskeletal or other disease-specific research, thus reinforcing the need for tailored communications about disease-specific research. Participants also emphasized the lack of trust in data sharing initiatives due to a lack of awareness (Textbox 2):

I don’t think the intricacies of it are well-known. I think people are still fearful of data sharing and it falling into the wrong hands...so they’re quite nervous about it still.

Interview 3, participant # F10

**Quotations representing awareness and motivations for supporting data sharing for musculoskeletal research.**

- “I think there are a lot of people who don’t understand it and are very suspicious of it.” [Interview 5, participant # M12]
- “I think it is a lot of unknown you don’t know who it is going to, where it is going to, what is going...” [Focus group 2, participant # F5]
- “I think if it was explained fully enough, as to why it was being used...look these are the benefits we’re using it for, this is what the potential could be. I think that would allay a lot of my fears, if I’m honest.” [Interview 10, participant # F17]
- “I don’t think people know that all the data’s being collected and you have to opt-out.” [Interview 12, participant # F23]
- “...my personal understanding would just simply have been my personal data, that would be shared but with my consent, where it’s needed to be shared.” [Interview 10, participant #F17]
- “it [data sharing] guides the researchers, it also helps innovation, invention and new methods of treatment can only be achieved with medical data.” [Focus group 4, participant # M25]
- “...the benefits would be that actually it’s going to improve healthcare, it’s going to improve diagnosis rates, it’s going to improve what offers are out there for support.” [Interview 10, participant # F17]
- “I’m happy for anyone to have my blood, it doesn’t matter who they are... it’s a very nice feeling... when people know the research they’re doing is helping other people, and in the way it’s helping other people and how things are being developed, I think that’s the positive side.” [Interview 16, participant # F31]

Before being informed about the Data Jigsaw program, few participants had awareness of the possibility that members of the public could opt out of having any of their personal health data shared by registering this choice via the NHS “opt-out” system. One participant who had previously worked for the NHS suggested that the opt-out had been kept a secret stating that, “I was there...for 20-odd years, I never knew that there was an opt-out...that’s a secret...I’m certainly not happy with that, that the opt-out is not transparent...I think it would be nice to have this option, I can’t understand why it’s hidden...” (Interview 13, participant # F24).

The discussions around opting out seemed to reflect some distrust of the NHS, making people feel disempowered about their choice to share data. There were also misunderstandings of opt-in and opt-out models of consent, suggesting there is a lack of awareness about the current opt-out model used by the NHS for research purposes with one of the participants stating that, “I know that there is an option in there where you can opt-in to share all of your medical records to be visible for research...” (Focus group 2, participant # F7).

It was recommended that the NHS opt-out be made more transparent, allowing the public the opportunity to opt-out if they wish to do so:

I think they (the public) need to be made aware that it happens, that it’s not freely available to pull out of it. It needs to be transparent I think; for people to be able to say no I’m not happy with that, don’t use my details et cetera.

Interview 13, participant # F24

In addition, there were some misunderstandings about how deidentified data can be used without individual consent for research purposes. Many presumed that individual consent was required stating, “I had always assumed, and I may be completely wrong that [data sharing for research] would require specific consent from the patient...” (Focus group 1, participant # F3).

This issue further highlights the lack of awareness regarding the opt-out consent model.

Although there was a general lack of awareness, many perceived data sharing for research positively, after being informed of the Data Jigsaw research program (Textbox 2). Data sharing for research was considered an opportunity to improve patient care. Participants voluntarily shared background experiences which prompted discussions about data sharing and the specific research being undertaken within the Data Jigsaw program. Some examples related to a project in the data pilot about the safety and effectiveness of analgesics, suggesting the clinical questions in the data pilot were of interest to the local population stating that “some of the medication...like non-steroidal anti-inflammatory drugs, I would like to hear more about them or how relevant it is to me, if it is good to me, you guys (researchers) will help me and tell me what more I need to do about it as well” (Interview 11, #M18).

Another respondent suggested medication safety was of paramount importance for patient benefit:

I personally am sensitive to opioids...a lot of the girls I’ve been speaking to on the [fibromyalgia blog]...not just in Salford, but they’ve been saying throughout hospitals in Manchester...a lot of the hospitals are taking people off the opioids because they think they’re being overused. They need to look into what type of ones are being given and how they are working, what are the benefits, what are not the benefits, how they work with...individual people.

Interview 6, participant # F13

For some, data sharing was compared with blood and organ donation and seemed to be viewed as a gift exchange that could benefit others. Thus, they felt that the benefits of health data sharing should be communicated more widely to establish trust. Examples, such as advertisements about organ donation were mentioned as models of building public trust:

[T]hey’ve got to promote the positive place of what comes out of this research and instances of how it supports people and how they can contribute to give people more confidence in the fact that it is being put to good use and that others will benefit as well as you. There’s an advert...on TV...and...it lists all the people (who benefit). Someone who got their eyes, somebody who got their heart, kidneys, tissue, so it’s the wider picture of what seems a simple instance of this is what can happen.

Interview 15, participant # F30

While many participants seemed motivated by the idea that musculoskeletal research could help them directly because of their personal experiences of musculoskeletal problems, this also highlighted altruistic motivations (ie, sharing data for the greater good and helping others). The above also indicates that learning from other campaigns may be beneficial and suggests that the impact of communication may be improved if they include motivation for supporting data sharing in this context.

A lack of awareness about the opt-out was also evident in the informal discussions with underserved communities, many of whom were not aware of this. One member had been notified of the NHS opt-out and chose to opt out of their data being used for research, noting their lack of trust in NHS organizations to keep data secure.

### Issues of Security and Sharing Data for Musculoskeletal Research Beyond the NHS

Privacy concerns remained a prevalent issue throughout the discussions. Some participants mentioned concerns regarding stigmatization stating that “there is so much fear about people being really scared of being judged and the stigma that comes along with if you’ve got a disability or if you’ve got a long-term health condition.” (Interview 12, participant # F23; see also Textbox 3).

**Quotations representing issues of security and sharing data for musculoskeletal research beyond the National Health Service.**

- “It can’t be emphasized enough how it [patient data] should be extremely secretive.” [Focus group 2, participant #M6]
- “I think if I had something that I was very mindful that I didn’t want other people knowing about me, I would be quite dubious...” [Interview 12, participant # F23]
- “[They] are a data management company...making algorithms and then feeding that back into the NHS...It’s not for their own benefits of...well, we’ve found this out and we’ll keep it to ourselves. It’s actually being fed back into the NHS to actually help the public...yeah, on that aspect...I would be inclined to go forth and let them have my data.” [Interview 3, participant # F10]
- “...people are really concerned...thinking about data generally how often it is sold for commercial use so I think that is the level of reassurance I certainly would want...that it suddenly wasn’t going to find itself with someone else who perhaps doesn’t follow the same ethical practices as the NHS...” [Focus group 1, participant # F3]
- “I think that would be for the good of everybody...and obviously they [external company] would be checked for security.” [Interview 2, participant # M9]

Discussions about the possibility of linking health and social care records for research indicated concerns about researchers accessing social care records, finding it intrusive stating that “...you feel like sometimes they pry a bit” (Interview 6, participant # F13). When discussing this issue in the context of a specific exemplar project, such as linking health and social care records to understand the impact of musculoskeletal problems [37], participants seemed positive:

[A] lot of stuff gets missed. There needs to be a better understanding. I think it is a need-to-know basis and if they need to know then it should be...there definitely needs to be a link between the social care and...rheumatology because again it’s getting back to this whole thing of, if they haven’t got the information they don’t know how to help you.

Interview 6, participant # F13

This suggests that participants were open to the idea of linking health and social care records once they became aware of the potential for patient benefit. Using a project-specific example also allowed the participants to consider whether it would be accessed on a “need to know” basis, establishing a basis for trust by providing transparent information about the benefits. This again strengthens the case for tailored communications about disease-specific research (rather than unspecified research) being conducted, for which there is then positive support.

Perspectives on sharing beyond the NHS to enable earlier diagnosis of musculoskeletal conditions, such as axial spondyloarthritis were shared. There were varying degrees of trust in commercial companies accessing patient data (Textbox 3):

I’m not sure about that...it can go a different way, can’t it? It’s all about the money, isn’t it, then...?

Interview 14, participant # M29

However, when discussing this issue in the context of a specific exemplar project, such as developing an algorithm for enabling earlier diagnosis [37], there were no objections from participants to data sharing for an external company in partnership with the university conducting the research. The partnership was viewed positively, on the condition their data were kept secure with participants stating “I don’t mind...as long as they keep...within the parameters of their research” (Interview 14, participant # M29).

Others drew on their struggles to attain a timely diagnosis and viewed the partnership positively, suggesting the Data Jigsaw research program was relevant to participants. This may have contributed to their positive views regarding collaborations with commercial companies. This may have also been influenced by the fact that the commercial company was working in partnership with trusted institutions, such as the NHS and an educational institution (Textbox 3):

I think that’s a great idea [to work with a private company to develop an algorithm to improve diagnosis]...in the early 80s...I started with a pain in my back...I was thrown from pillar to post, I was given pain killers...I had x-rays and they said I had this spondylarthritis...that went on for several years...it was the early 2000s when I had an actual body scan of my back...to be told that it was too late, they couldn’t do anything. So, with everything you’ve just said to me, that could have been avoided...

Interview 13, participant # F24

Individuals seemed to trust the NHS to keep their data secure, however, they noted the importance of improved transparency regarding external collaborations, to build public trust. It was recommended that a private company’s credentials be made transparent, suggesting trust can be established if the company is perceived as reputable and has a positive track record of working for patient benefit, the latter being one of the reported motivations for participants to share data:

I’d want to know about the private company and their background. How legit are they? We do trust the NHS. Private companies are popping up all over the place, it could be a private company from abroad wanting that data. So, you’d be like oh, put the brakes on. What’s happening here? Why’s a private company wanting that? What’s their past? What have they done in the past for research and patients? You’d need to be able to backup who they actually were. They’d have to have credentials.

Interview 3, participant # F10

The concerns around privacy and discrimination were also apparent in informal community discussions. Such discussions highlighted a lack of trust in commercial companies and concerns that sensitive information may be shared with others and potentially used against them; for example, in relation to judgments about their parenting capacity. However, they were also supportive of the specific projects described as part of the Jigsaw program and related these to potential benefits relevant to their own musculoskeletal problems.

### Transparency and Communication Needs for Building Trust in Data Sharing for Musculoskeletal Research

Participants expressed a need for different ways to raise awareness and build trust within affected communities about health data sharing for musculoskeletal research. Transparency about data security and governance was considered vital to building trust (Textbox 4). A key message to relay to the public was that their data was being used for research and that it was kept safe with one of the participants stating that “The message you need to bang home is that it is only going to be for research, and it is safe” (Focus group 1, participant # F3).

**Quotations representing transparency and communication needs for building trust in data sharing for musculoskeletal research.**

- “[W]hat you want to do is reassure people that notwithstanding that they have got your information there really is no problem to you as a member of the public.” [Focus group 1, participant # F3]
- “I think it is good for people to hear how their data is being used, how it is being stored and all of those procedures...” [Focus group 2, participant # F5]
- “[F]or me it would be where is my information going, who is going to use it and how are they going to use it.” [Focus group 2, participant # F5]
- “[I]f you are delivering that message more generally it will need to be a little more digestible about how you are managing the data...although you are identified by your NHS number it still isn’t really a threat to you personally, there is not going to be a great leakage of your personal data so I think that is something that you could probably work on.” [Focus group 1, participant # F3]
- “[P]eople who are hesitant about, I don’t want to share the information, I don’t want my information used...it may be reassuring to them to see that this is secure, it’s been deidentified, that if you choose you could opt out but here’s the kind of way it’s being used and how much good it can do.” [Focus group 6, participant # F45]
- “[I]t’s better to have the findings and sell the positive message of we have done this and this is what you have told us and we are thinking about this.” [Interview 16, participant # F31]

Although the majority expressed a view to raise awareness about data security protocols, a few individuals suggested that it is quite complex to understand and might cause confusion, suggesting it is important to consider the layers of information required to engage the public without added complexity (Textbox 4) with statements such as “it just needs to be short and snappy but not overburdened with—I think one of the terms used was locked box and whilst I know what a locked box is, I...wonder is that terminology describing a process that is used internally that actually somebody outside of that might not understand” (Focus group 2, participant # F7).

In addition, to establish trust, it was considered crucial to promote the collaborative relationship between the educational institution involved in the Data Jigsaw research program [35] and the NHS, reflecting the public’s social investment in support of the NHS for the greater good. The short films used in this study demonstrated a strong link between the two. One of the participants stated that “with this group, a lot of the researchers are either still practicing clinicians or there is a strong link between the educational institution and the NHS trust there is some kind of positive relationship that isn’t there for financial gain, it is there for the advancement of scientific knowledge for research helping the development of new treatments” (Focus group 2, participant # F7).

When asked if they would like information about any research projects using their health data, participants were overwhelmingly positive. One respondent suggested producing a summary report that could be circulated and to demonstrate this, used an example of donating to charity where donors are updated about their contributions. They said that “if you donate to Oxfam you get a letter each year saying your goods have been sold for this amount of money and it just makes you think that some use has come out of you donating your time if you like to the research...” (Focus group 1, participant # F3).

This suggests that receiving information about how patient data has been used could build and sustain public trust and echoes the patient’s willingness to be acknowledged as an active agent in data sharing initiatives [42].

Research participants highlighted the importance of accessible communications as well as the importance of actively engaging with communities:

[W]e’re being challenged, rightly so, by communities saying, you’re not getting information out to us, it’s a one-way process, it’s not a discussion...it’s really about valuing those community links we’ve got...You’ve got to make sure...that the information that’s going out is what you want the information to say...

Interview 12, participant #F23

Others reiterated that “community service” is imperative to raising awareness (Interviews 17 and 18, participants #M32 and #M33).

During discussions with members of underserved communities, the issue of digital exclusion was raised. People highlighted the perceived lack of engagement and suggested researchers commit to ongoing dialogue with local communities by regularly visiting community spaces in-person and speaking directly to those who may be impacted by health data sharing and valuing community links.

This highlights the importance of digital inequalities and the need to consider digital inclusion as part of transparency and communication needs to ensure information is accessible and includes a balance of audio-visual communications and in-person discussions.

## Discussion

### Principal Findings

This study used examples from a local data integration pilot for arthritis research (known as the Data Jigsaw) [37] to explore participants’ views about health data sharing for musculoskeletal research. It explored ways to improve transparency and enable dialogue with local communities about health data sharing for research, to inform the design and delivery of better ways of communicating and building public trust. Three key themes characterized participants’ views: awareness and motivations for supporting data sharing for musculoskeletal research, issues of security and sharing data beyond the NHS for musculoskeletal research, and transparency and communication needs for building trust in data sharing for musculoskeletal research. The discussion is centered on the 3 key themes below.

### Awareness and Motivation for Supporting Data Sharing for Musculoskeletal Research

In keeping with previous studies [4,6,8], a general lack of awareness about data sharing for research was evident among participants. The responses also resonate with previous research suggesting patients with musculoskeletal problems adopt an altruistic attitude toward helping others and a sense of social responsibility to share data for the greater good, along with personal motivations to share data for self-benefit [4]. However, previous research and communication materials to inform the public about health data sharing has often focused at a more general level rather than specific conditions within local contexts [9]. After being informed of specific examples of research within the Data Jigsaw program, people expressed positive views about sharing health data for musculoskeletal research. Participants often related discussions about data sharing in this context to their own experiences of musculoskeletal conditions and potential benefits for themselves as well as others. This highlights the potential value of tailoring specific information for a local and condition-specific population to enable meaningful engagement and greater awareness and support for specific research.

In line with previous studies [16,17], there were misunderstandings of opt-in and opt-out models of consent due to limited awareness. This meant that few participants were aware of the national data opt-out as has been found in previous studies [43], with participants suggesting it was hidden from them. This suggests education about the national data opt-out is required and that it is important to notify patients about the opportunity to opt out if they wish to do so.

Respondents believed sharing specific examples of the potential for patient benefit from health data sharing was important to gain public trust. They provided examples of mass advertising campaigns as exemplar models of establishing public trust. Learning from other campaigns may be considered beneficial and suggests that communication and trust may be improved if such campaigns reflect the public’s motivation for supporting data sharing, such as reflecting on the public’s sense of social responsibility to help others. Currently, there are minimal opportunities for members of the public to learn more about how their health data are used for research [43], a finding that our research supports. Promoting the benefits of data sharing, as well as exploring a range of ways to disseminate the research outcomes and the national opt-out may help garner further support. Examples might include developing a range of resources to interact with the public, such as posters and leaflets; or using social media or websites; and speaking to local community members to increase awareness. Organizations like Understanding Patient Data [44] have done this well, developing various resources about health data sharing for research. Dissemination of the research outcomes via public events may also attract attention and help establish public trust. It is recommended that wider advertising both to inform the public about the national opt-out, the patient benefits of health data sharing, and a thank-you for taking part may help build public trust.

### Issues of Security and Sharing Data Beyond the NHS for Musculoskeletal Research

Similar to previous studies [4,6,9,43], respondents held positive views toward the NHS and educational institutions collaborating to improve patient care. There were issues of data security when sharing data beyond the NHS, particularly when accessing sensitive information that could potentially be used against them in wider aspects of life, including social care records and the involvement of private companies. Conversely, respondents held positive views when discussing data sharing in the context of specific exemplar projects in the Data Jigsaw program. Their comments suggest the project-specific exemplars may have influenced participants’ views toward data sharing for research once they became aware of the potential for patient benefit.

The principles of data minimization (ie, accessing data by trained individuals and not sharing data more widely), were also highlighted, suggesting transparency about who has access to the data can help increase the acceptability of the reuse of health data for research and establish public trust [43]. This aligns with the SDE model [35] and has been applied in the Data Jigsaw program [37]. The findings in this study reflected positive attitudes toward collaborations with external companies to improve patient care, such as improving diagnosis. This relates back to participants’ reported motivations for data sharing, as it seemed participants viewed external collaborations positively if the company had an altruistic motive to benefit patient care. This again draws on the public’s motivations and sense of social responsibility [4] to share data for personal and community benefit and suggests views may be influenced depending on the partnerships commercial companies have with trusted institutions, such as the NHS. These findings emphasize the potential benefits of providing specific information regarding collaborations with external companies, as well as the company’s ethos of working for patient benefit to garner further support. In addition, including details of any potential collaborations with trusted institutions, such as the NHS, and describing who has access to data may mitigate the public’s security concerns (ie, whether an external company has access to patient data). Researchers can also provide a data access report or data receipt, providing clarity about who has accessed their data.

Although public notification might be considered one way of garnering positive support, it is important to consider that campaigns rely on resources and funding. Mass advertising campaigns can be well-funded and effective, however, those with limited resources may suffer. It may be necessary to explore how ethical and governance processes can be made robust and transparent to ensure that the public is aware about of how their data are used without relying on mass advertising to improve awareness.

### Transparency and Communication Needs for Building Trust in Data Sharing for Musculoskeletal Research

The findings suggest participants valued transparency about data security, the national data opt-out, and research outcomes. Various ways about how best to communicate with the wider public about data sharing in local communities were suggested, such as developing simple and layered communications with accessible messages. Participants also gave positive feedback on audio-visual resources and summaries used to describe examples from the Data Jigsaw program. The results of this study have been used to inform a public notification campaign, the development and outcome of which will be reported separately.

The findings highlighted a lack of awareness and misunderstanding regarding security and governance of the use of personal health data for research and identified a need to fill a gap in resources to explain SDEs. In line with the Goldacre review’s recommendations to be transparent about how data are used [31], participant views informed the development of a short film to explain SDEs in a creative and understandable way for use in a public notification campaign for the Jigsaw program [45]. Raising awareness about the national data opt-out was also considered and implemented via the study website [37].

Our study maximized the inclusion of diverse communities, both through diversity among research participants and public involvement and engagement. This is important as literature has pointed to lower trust in data sharing among British ethnic minorities [23,24] and the need for greater inclusion of seldom heard voices to be part of public dialogue and governance associated with data sharing [25]. Feedback from diverse participants indicated that ongoing dialogue and engagement by reporting research outcomes through various methods, such as visiting community spaces in-person can be implemented to improve transparency and build and sustain public trust in local communities. Previous research has noted that fostering engagement and open dialogue with those who may be directly affected by data sharing may be considered challenging for researchers, but this is important for enabling support for data sharing and for sustaining public trust [42]. It also places emphasis on the importance of reciprocity and coproduction, enabling participants to be active agents in the patient-researcher relationship [42].

### Strengths and Limitations

This study further explores issues raised in previous research within general population contexts [4,6,8,9,43] with the benefit of having a specific clinical context (ie, musculoskeletal research). This adds to previous work to raise awareness and collaboration on data sharing with patients affected by specific conditions, such as cancer [36] or genetic conditions [46]. The specific clinical focus with clearly specified research questions is considered a strength in being able to study perspectives and information needs directly with affected communities.

A key strength has been our extensive and inclusive approach to ensure public involvement and engagement has been embedded throughout the research, including a small coproduction working group and our wider public engagement using informal discussions in community-based settings. Speaking to people informally and in-person enabled wider engagement with the public, particularly those without access to information technology, such as members of underserved communities. The value of an inclusive and informal approach enabled relaxed discussion and communication about data sharing within busy community spaces where people already meet informally, enhancing the breadth of perspectives included in this research and providing an example of how other research studies might include communities who have previously been labeled as being “hard-to-reach” [24].

Conducting focus groups and interviews on the web via Zoom presented unique challenges. While most participants opted to use their video cameras, a small number used the camera minimally. For example, some members introduced themselves at the start with the camera on but then turned the camera off for periods of the discussion. In such cases, this seemed to alter the group dynamics and brought some challenges for enabling dialogue and discussion. This required active management by researchers to ensure all members felt included and maintain the momentum of discussion as recognized in previous research [47].

### Conclusions

Views of participants in this research were generally positive about the sharing of health and social care data for musculoskeletal research purposes but there were also low levels of awareness regarding processes and governance of health data sharing. There were associated views about the need for transparency about data security and how patient data would be used to inform patient benefit, reflecting similarities in wider literature [4,6,15-18]. This study used project-specific examples from a local data integration pilot for arthritis research (known as the Data Jigsaw) and found that participants with musculoskeletal conditions (or at risk of such conditions) were supportive of examples discussed when details were explained, the purpose of analysis, the partnerships entailed, how data would be kept secure, and specific potential benefits for people with musculoskeletal conditions. The findings have important implications for research policy and practice. Building and sustaining public trust has been identified as crucial for enabling health data sharing for research. However, few studies have included a focus on understanding and building public trust [9]. This paper adds novel insights by focusing on how to build public trust based on the perspectives of people with relevant musculoskeletal problems within a local area where health records were being used for musculoskeletal research. The combination of public involvement combined with qualitative research was found to be extremely valuable for a more inclusive approach [48,49] similar to other studies in the digital health field. In addition, the wider community-based informal discussions enabled wider inclusion of “seldom heard” voices considered vital for building public trust in health data sharing. Our findings indicate that transparency, and tailoring communications to reflect current and local research using health records, can help to raise awareness and build public trust and support in health data sharing for musculoskeletal research. As done for the Jigsaw program [37], we recommend that researchers using health data records should include a direct weblink to the NHS opt-out on communications, such as a poster or leaflet or a dedicated website to the research itself. The project also highlights the value of engagement within community settings, in addition to audio-visual materials for enabling inclusive dialogue that may contribute to building public trust in data sharing. It is recommended that researchers using data from shared health care records include adequate resource patient and public engagement in their project plans from the onset of the research.

## Appendix

Multimedia Appendix 1 Topic guide for focus groups and interviews.

Multimedia Appendix 2 Completed COREQ (Consolidated Criteria for Reporting Qualitative Research) checklist.

Multimedia Appendix 3 Completed Guidance for Reporting Involvement of Patients and the Public (GRIPP2) checklist.

## Abbreviations

COREQ: Consolidated Criteria for Reporting Qualitative Research
GRIPP2: Guidance for Reporting Involvement of Patients and the Public
NHS: National Health Service
PPIE: patient and public involvement and engagement
SDE: Secure Data Environment
