# Supplementary material for: Building and Sustaining Public Trust in Health Data Sharing for Musculoskeletal Research: Semistructured Interview and Focus Group Study
Source: J Med Internet Res. 2024 Oct 15;26:e53024. doi: 10.2196/53024 (PMC11522652; doi:10.2196/53024)
Supplement: Multimedia Appendix 1 [file jmir_v26i1e53024_app1.pdf]

Appendix 1. Topic guide for focus groups and interviews

“Assembling the Data Jigsaw”

Understanding and building public and patient trust in health data sharing for research to improve diagnosis, treatment and services for people with musculoskeletal conditions

Introduction

Thank you for participation and consent will be covered prior to commencing interview

Reassurance of confidentiality

Enabling all participants to contribute

Any questions before we start

Initial intro question – Introduce ourselves, and say why you are interested in this topic

A summary of the JIGSAW programme – aims and reasons for doing this work (short film – final version of the film is available here https://vimeo.com/814636851)

Any questions about this?

A summary of the work-package focusing on building and sustaining public trust

*We know that many people are supportive of their heath data being used for research with personal identifiable information removed

*However, we also know there is little awareness about how health data is already used for research and the challenges of improving how it is used

*Public trust is crucial for enabling this kind of research to be done and to improve awareness and trust regarding health data sharing there needs to be opportunities for discussions with public and patients. We also need to develop better ways of communicating information about research using health data

*These are the issues we want to discuss with you today. We would like to ask a few questions about your views on sharing health data for research? What information do you think people need, and how can we enable better communication? How can we create simple messages to raise awareness about this topic and for the research being done in Salford using health data for MSK research?

Awareness of, and general views on current arrangements for storing and viewing health information.

- What data sharing means to them, if anything at all, or what they understand by data sharing (generally, or in health context).

- How much were you aware of how personal health data is stored and shared for research before taking part in this study – has awareness of use of health data changed over time? How it is used

- Ask if they would like to view a short 2 minute film about data sharing to summarise how personal health data is stored and used

    - A 2 minute video presentation to introduce how data is kept safe based on the principles of the 5 safes framework (see published version of the video at the following web link (https://thedatajigsaw.co.uk/)

- What are your views about any of the benefits or potential problems with health data sharing for research?

    - Do you know how to access your health records? Have you ever accessed your personal health records before?

- How much does it matter to you to know how your details are stored and shared?

    -Why/Why not?

- Do you have any concerns about who has access to your personal health information and data?

- Have you got any examples of when your data has been shared and it has improved your healthcare experience, access to healthcare or social care services?

- Have you got any examples of when your data has not been shared in a healthcare setting and it has impacted your healthcare experience, access to healthcare or social care services?

- What do you think are the potential benefits for people sharing their personal health information with researchers?

- How much trust do you have in the NHS or research organisations (i.e. Universities) electronic security systems to protect your privacy?

Examples of research being conducted within the Data JIGSAW programme (see the project summaries for the work packages on the following web page https://blogs.manchester.ac.uk/centre-for-epidemiology/assembling-the-data-jigsaw/)

Present one or two examples based on plain language summaries or short film (all films were video versions of the project summary read by a member of the research team).

What are your views about this work?

Any questions? Is this easy to understand? How could the information be improved?

Your views on how best to explain and communicate about the JIGSAW programme

Next we would like to show some images and messages that have been co-developed with our public advisory group and ask your views about these.

Show images on screen / handouts

Image/ caption 1.

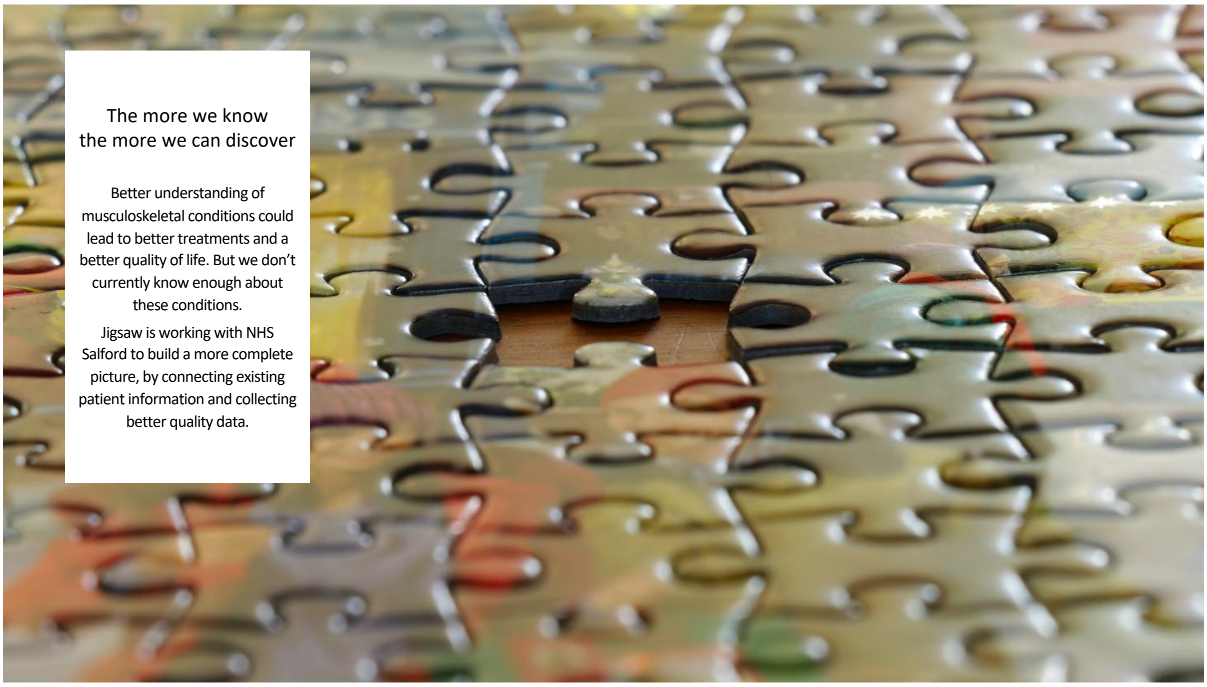

Image / caption 2

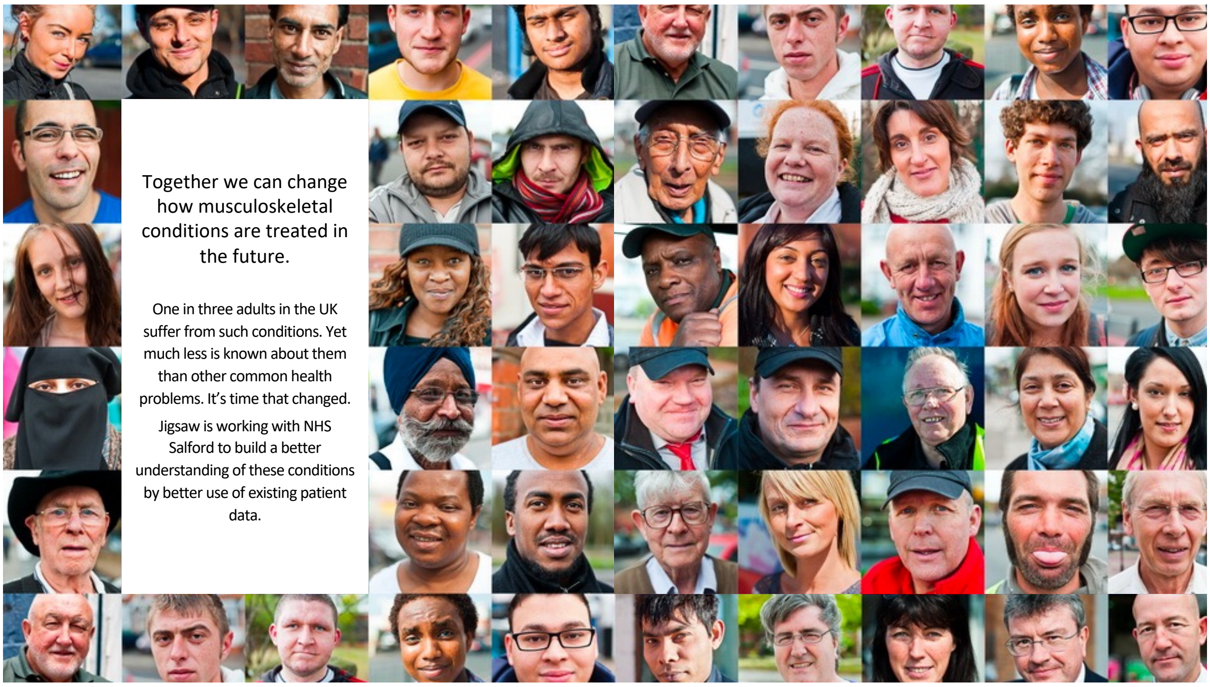

Image/ caption 3

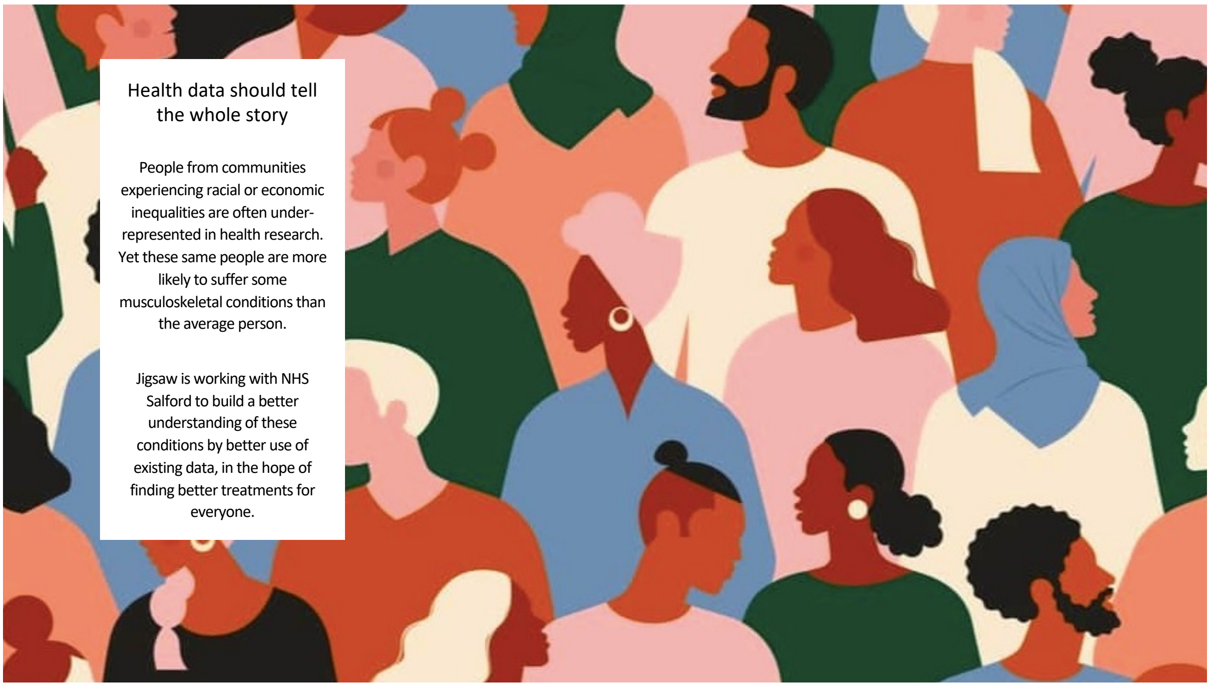

Image/ caption 4

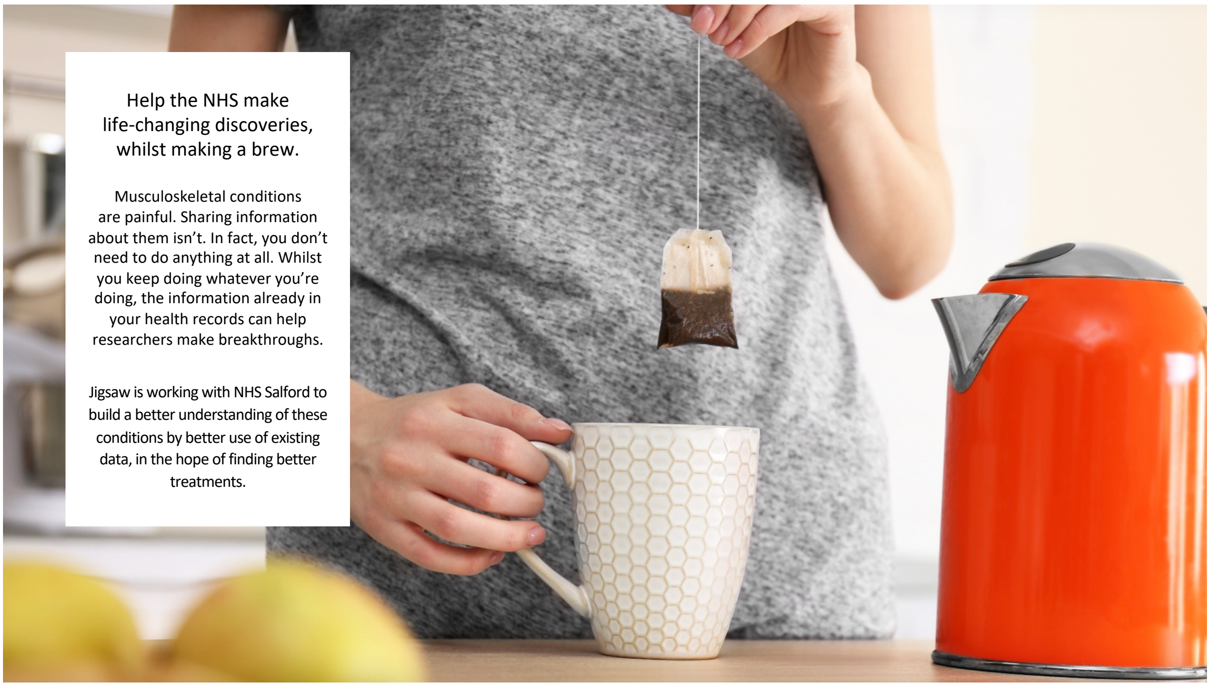

Follow-up focus groups only

For follow-up focus groups the above images and caption were replaced with the finalised designs as used for a website designed for the study (see the study website via the following link https://thedatajigsaw.co.uk/) and in a communications campaign where posters were displayed on bill-boards and at local bus stops.

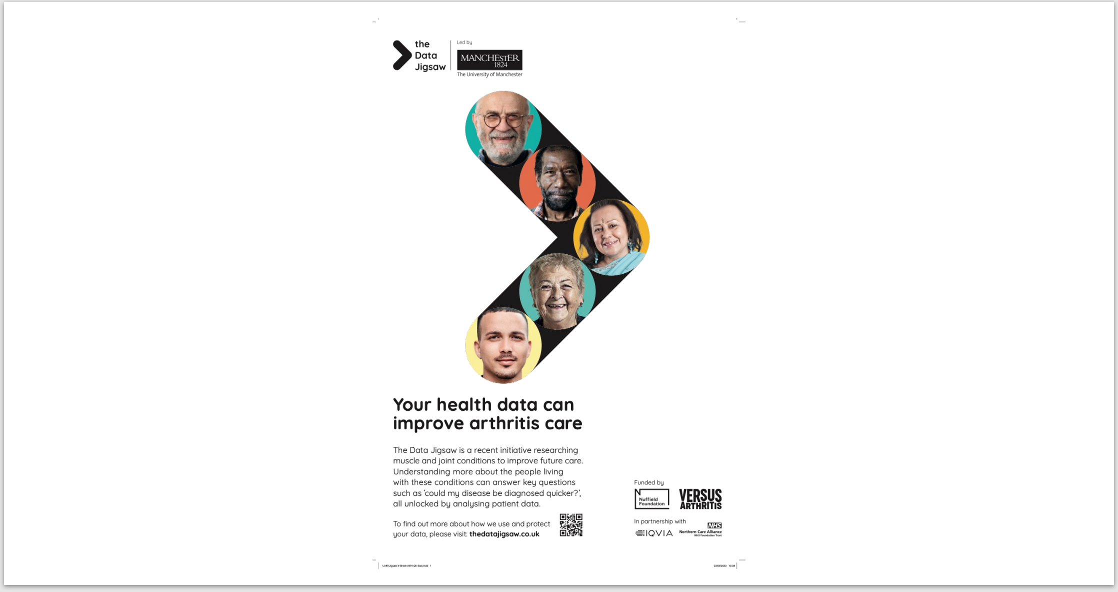
the Data Jigsaw
Led by
MANCHESTER 1824
The University of Manchester
Your health data can improve arthritis care
The Data Jigsaw is a recent initiative researching muscle and joint conditions to improve future care. Understanding more about the people living with these conditions can answer key questions such as 'could my disease be diagnosed quicker?', all unlocked by analysing patient data.
To find out more about how we use and protect your data, please visit: thedatajigsaw.co.uk
Funded by
Nuffield Foundation
VERSUS ARTHRITIS
In partnership with
IQVIA
NHS
Northern Care Alliance
NHS Foundation Trust

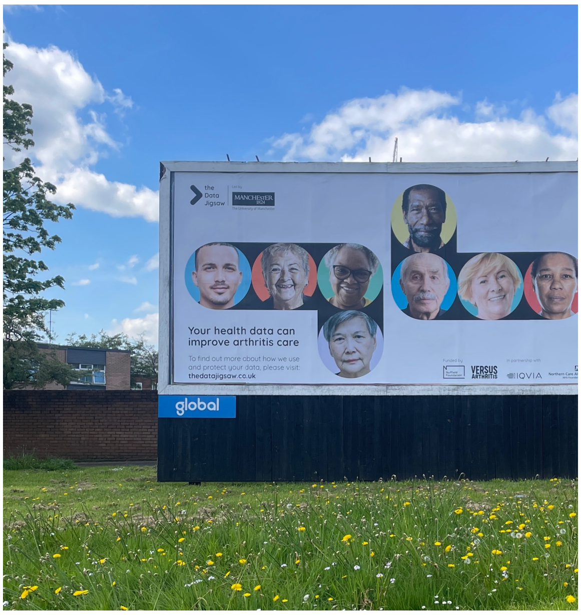
the Data Jigsaw
Your health data can improve arthritis care
global

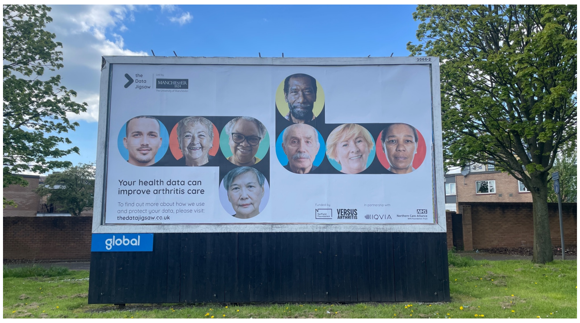
Your health data can improve arthritis care
global

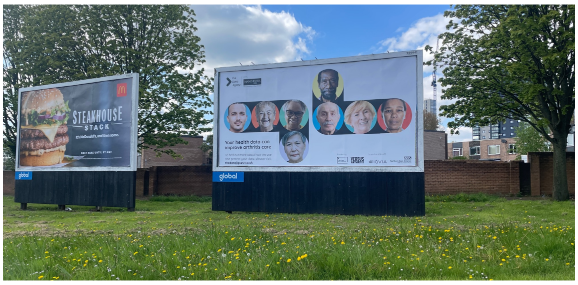
STEAKHOUSE STACK
global
