# Supplementary material for: Building and Sustaining Public Trust in Health Data Sharing for Musculoskeletal Research: Semistructured Interview and Focus Group Study
Source: J Med Internet Res. 2024 Oct 15;26:e53024. doi: 10.2196/53024 (PMC11522652; doi:10.2196/53024)
Supplement: Multimedia Appendix 3 [file jmir_v26i1e53024_app3.pdf]

GRIPP2 short form

Section and Topic	Item	Reported on page number
1. Aim	**Report the aim of the PPI within the study**<br><br>The aim of working with the PPIE group was to enable public involvement in all stages of the qualitative research and the co-production of communications materials in order to inform patients and public of research being conducted using local health care records. The latter was part of a wider research programme known as 'The Data Jigsaw'<br><br>We also aimed to enable wider engagement with affected communities, particularly with members of underserved groups. This was to maximise inclusion of a diverse range of public and patients. The informal discussions supported wider public dialogue regarding the Data Jigsaw research programme and also enabled further input into the design of the qualitative research and development of communications for a public notification campaign.	4<br><br>5
2. Methods	**Provide a clear description of the methods used for PPI within the study**<br><br>Authors ZK Yusuf and C Sanders met with the PPIE group quarterly to engage with them about different aspects of the study, such as the design of participant information sheets, interview / focus group topic guides, and communications materials explaining health data use. We also worked together with the PPIE group to discuss and refine emerging findings from the interviews and focus groups.<br><br>Wider community engagement discussions included local members of the public who may not have had the opportunity to express their opinions about health data sharing previously and included people at risk of poverty and homelessness (via a community-based drop-in centre), as well as members of the D/deaf community. With the help of the PPIE advisory group, authors ZK Yusuf and C Sanders arranged to attend a drop-in centre for people at risk of homelessness on two occasions to generate interest. Two informal discussions were then arranged and took place at the community-based drop- in centre with approximately 32 people. Further, ZK Yusuf liaised with community gatekeepers to arrange informal discussions	4<br><br>5

	with the D/deaf community (n=15) and with volunteers who were part of a community allotment group (n=24). Approximately 71 people took part in the informal discussions.<br><br>Informal discussions took place at the same time as the formal focus groups and interviews with research participants. The informal discussions were not recorded and no personal data were collected. The ethics committee approved plans for public involvement and wider community engagement. However, formal recorded consent was not required as part of the ethical governance of the study because attendees at informal discussions were not research participants. An introduction to the research topic and the purpose of the discussion was provided at the start of the discussion. It was made clear that the discussion was informal and that we would not be recording any identifiable information and would not be audio-recording the discussions. Similar to the research focus groups, open questions were asked about views and understanding of using health records for research. Brief notes were created during and following the discussions but without any personal identifiers. The notes were in bullet point form in order to identify prevalent issues. Whilst the informal discussions were not part of the qualitative analysis, we have referred to these where there was resonance with analytic themes. This serves to reflect how public involvement and engagement was embedded throughout.	6
3. Study results	**Outcomes – report the results of PPI within the study, including both positive and negative outcomes**<br><br>The detailed activities and outcomes of our PPIE work is being reported in a distinct PPIE focused paper. However, we have summarised some of the key results of the community engagement work within the current paper.<br><br>The core PPIE group made extensive contributions to the design of study materials and improved the language and design of audio-visual materials to reflect key messages in a simple and accessible way for diverse communities.<br><br>Discussions within the wider community engagement groups resonated with some of the discussions completed within the research focus groups, but highlighting examples particular to their own context.	

	Key issues raised included the lack of awareness about security of data used for health research. One member had been notified of the NHS opt-out and chose to opt out of their data being used for research, noting their lack of trust in NHS organisations to keep data secure.<br><br>The concerns found within the research regarding privacy and discrimination were also apparent in informal community discussions. Such discussions highlighted lack of trust in commercial companies and concerns that sensitive information may be shared with others and potentially used against them; for example, in relation to judgements about their parenting capacity. However, they were also supportive of the specific projects described as part of the JIGSAW programme and related these to potential benefits relevant to their own musculoskeletal problems.<br><br>During discussions with members of underserved communities, the issue of digital exclusion was raised. People highlighted the perceived lack of engagement and suggested researchers commit to ongoing dialogue with local communities by regularly visiting community spaces in-person and speaking directly to those who may be impacted by health data sharing and valuing community links.	11<br><br>13<br><br>15
4. Discussion and conclusions	**Outcomes – comment on the extent to which PPI had an impact on the study overall. Describe positive and negative effects**<br><br>Importantly, there was emphasis on enabling dialogue and engagement with diverse communities, particularly for members of marginalised groups who may have limited access to information technology (IT) and other common forms of communication such as social media. Enabling ongoing dialogue and engagement by reporting research outcomes through various methods, such as visiting community spaces in-person can be implemented to improve transparency and build and sustain public trust in local communities. Fostering engagement and open dialogue with those who may be directly affected by data sharing may be considered challenging for researchers, however it acknowledges the important role that local communities play in the data sharing research process and may be significant for sustaining public trust. It also places emphasis on the importance of reciprocity and co-production, enabling participants to be active agents in the patient-researcher relationship.	

	A key strength has been our extensive and inclusive approach to ensure public involvement and engagement has been embedded throughout the research, including a small co-production working group and our wider public engagement using informal discussions in community-based settings. Speaking to people informally and in-person enabled wider engagement with the public particularly for those without access to information technology such as members of underserved communities. The value of an inclusive and informal approach enabled relaxed discussion and communication about data sharing within busy community spaces where people already meet informally, enhancing the breadth of perspectives included in this research and providing an example of how other research studies might include communities who have previously been labelled as being 'hard-to-reach' [24].<br><br>the wider community based informal discussions enabled wider inclusion of 'seldom heard' voices considered vital for building public trust in health data sharing.<br><br>the wider community based informal discussions enabled wider inclusion of 'seldom heard' voices considered vital for building public trust in health data sharing.	
5. Reflections/ critical perspective	**Comment critically on the study, reflecting the things that went well and those that did not, so others can learn from this experience**<br><br>We worked closely with a PPIE co-investigator and our core group at the outset of the study in designing a strong PPIE strategy that placed this centrally for our programme and was hugely beneficial. We also received good feedback from members.<br><br>The wider engagement with community groups for informal discussions worked very well in enabling maximum inclusion and public dialogue regarding the research with diverse communities.<br><br>Problems we encountered included losing a key member of our PPIE group early in the research who needed to withdraw due to personal reasons. However, the remaining members of the group continued to give input and helped facilitate connections with wider community groups such as enabling the informal discussion group within the D/deaf community.	

	Whilst the extensive community engagement conducted for this programme was hugely valuable with >70 public members. However, we recognise that this is also costly in order to ensure public members are financially reimbursed and supported to take part.	
